# United Kingdom Diabetic Retinopathy Electronic Medical Record (UK DR EMR) Users Group: report 4, real-world data on the impact of deprivation on the presentation of diabetic eye disease at hospital services

**DOI:** 10.1136/bjophthalmol-2018-312568

**Published:** 2018-09-29

**Authors:** Alastair K Denniston, Aaron Y Lee, Cecilia S Lee, David P Crabb, Clare Bailey, Peck-Lin Lip, Paul Taylor, Maria Pikoula, Esther Cook, Toks Akerele, Richard Antcliff, Christopher Brand, Usha Chakravarthy, Randhir Chavan, Narendra Dhingra, Louise Downey, Haralabos Eleftheriadis, Faruque Ghanchi, Rehna Khan, Vineeth Kumar, Aires Lobo, Andrew Lotery, Geeta Menon, Rajarshi Mukherjee, Helen Palmer, Sudeshna Patra, Bobby Paul, Dawn A Sim, James Stephen Talks, Elizabeth Wilkinson, Adnan Tufail, Catherine A Egan

**Affiliations:** 1 University Hospitals Birmingham NHS Foundation Trust, Birmingham, UK; 2 University of Birmingham, Birmingham, UK; 3 NIHR Biomedical Research Centre at Moorfields Eye Hospitals NHS Foundation Trust, University College London Institute of Ophthalmology, London, UK; 4 University of Washington, Seattle, Washington, USA; 5 City University London, London, UK; 6 University Hospitals Bristol NHS Foundation Trust, Bristol, UK; 7 Birmingham and Midland Eye Centre, Sandwell and West Birmingham Hospitals NHS Trust, Birmingham, UK; 8 Institute of Health Informatics, University College London, London, UK; 9 East Kent Hospitals University NHS Foundation Trust, Kent, UK; 10 Hinchingbrooke Health Care NHS Trust, Hinchingbrooke, UK; 11 Royal United Hospital Bath NHS Trust, Bath, UK; 12 Sheffield Teaching Hospitals NHS Foundation Trust, Sheffield, UK; 13 Department of Ophthalmology, Royal Victoria Hospital, Belfast, UK; 14 Mid Yorkshire Hospitals NHS Trust, Wakefield, UK; 15 Hull and East Yorkshire Hospitals NHS Foundation Trust, Hull, UK; 16 King's College Hospital NHS Foundation Trust, London, UK; 17 Bradford Teaching Hospitals NHS Foundation Trust, Bradford, UK; 18 Calderdale and Huddersfield NHS Foundation Trust, Calderdale, UK; 19 Wirral University Teaching Hospital NHS Foundation Trust, Wirral, UK; 20 Moorfields Eye Centre at Bedford Hospital, Bedford, UK; 21 Faculty of Medicine, University of Southampton, Southampton, UK; 22 Frimley Park Hospital NHS Foundation Trust, Frimley, UK; 23 Leeds Teaching Hospitals NHS Trust, Leeds, UK; 24 Barts Health NHS Trust, London, UK; 25 Barking, Havering and Redbridge University Hospitals NHS Trust, Romford, UK; 26 Moorfields Eye Centre at Croydon University Hospital, London, UK; 27 The Newcastle Upon Tyne Hospitals NHS Foundation Trust, Newcastle, UK; 28 Northern Devon Healthcare NHS Trust, Barnstaple, UK

**Keywords:** electronic medical record, diabetes

## Abstract

**Aim:**

To assess the impact of deprivation on diabetic retinopathy presentation and related treatment interventions, as observed within the UK hospital eye service.

**Methods:**

This is a multicentre, national diabetic retinopathy database study with anonymised data extraction across 22 centres from an electronic medical record system. The following were the inclusion criteria: all patients with diabetes and a recorded, structured diabetic retinopathy grade. The minimum data set included, for baseline, age and Index of Multiple Deprivation, based on residential postcode; and for all time points, visual acuity, ETDRS grading of retinopathy and maculopathy, and interventions (laser, intravitreal therapies and surgery). The main  outcome measures were (1) visual acuity and binocular visual state, and (2) presence of sight-threatening complications and need for early treatment.

**Results:**

79 775 patients met the inclusion criteria. Deprivation was associated with later presentation in patients with diabetic eye disease: the OR of being sight-impaired at entry into the hospital eye service (defined as 6/18 to better than 3/60 in the better seeing eye) was 1.29 (95% CI 1.20 to 1.39) for the most deprived decile vs 0.77 (95% CI 0.70 to 0.86) for the least deprived decile; the OR for being severely sight-impaired (3/60 or worse in the better seeing eye) was 1.17 (95% CI 0.90 to 1.55) for the most deprived decile vs 0.88 (95% CI 0.61 to 1.27) for the least deprived decile (reference=fifth decile in all cases). There is also variation in sight-threatening complications at presentation and treatment undertaken: the least deprived deciles had lower chance of having a tractional retinal detachment (OR=0.48 and 0.58 for deciles 9 and 10, 95% CI 0.24 to 0.90 and 0.29 to 1.09, respectively); in terms of accessing treatment, the rate of having a vitrectomy was lowest in the most deprived cohort (OR=0.34, 95% CI 0.19 to 0.58).

**Conclusions:**

This large real-world study suggests that first presentation at a hospital eye clinic with visual loss or sight-threatening diabetic eye disease is associated with deprivation. These initial hospital visits represent the first opportunities to receive treatment and to formally engage with support services. Such patients are more likely to be sight-impaired or severely sight-impaired at presentation, and may need additional resources to engage with the hospital eye services over complex treatment schedules.

## Introduction

Unequal distribution of wealth and other resources occurs in almost all societies.^1 2^ The WHO have outlined how health inequality may arise from differences in socioeconomic position.^3^ It is recognised that this inequality leads to ‘deprivation’ in a proportion of society, and that this is not solely economic, but may include limited access to resources for cultural, social, knowledge or political reasons.^2 4^ In the UK, the report *Fair society, healthy lives* (also known as ‘the Marmot review’ after its author) highlighted the extent to which health inequalities exist in the UK, and the profound impact of income deprivation on life expectancy and disability-free life expectancy. Although the influence of deprivation on health was already widely accepted, the Marmot review brought this issue firmly to the modern political consciousness of the UK.^5 6^

Quantifying this effect allows agencies to measure the greatest areas of unmet need, and to target resources to deal with specific deprivation-related barriers to healthcare. Since 2000, the UK government has been measuring deprivation in England using the Index of Multiple Deprivation (IMD). The IMD splits deprivation into discrete, quantifiable domains, including income, employment, health and disability, education, crime, barriers to housing, services, and living environment. These are collated at the level of small geographical areas known as lower layer super output areas (LSOAs), enabling deprivation to be estimated for an individual according to their residential area. IMD data have successfully been used to highlight social deprivation as an independent risk factor for many systemic diseases.^5^ In terms of eye disease, the IMD has been used to evaluate the impact of deprivation on low vision^7^ and a significant factor in the prevalence or presentation of a number of specific eye conditions, including severe neovascular age-related macular degeneration,^8^ glaucoma^9 10^ and cataract.^11^

In the global setting a number of studies have explored the potential influence of deprivation on the prevalence of diabetes, access to assessment and treatment, quality of glycaemic control, and diabetic complications; this has been evaluated in systematic reviews by Lindner *et al* for type 1 diabetes (2018) and Grintsova *et al* for type 2 diabetes (2014).^12 13^ In terms of diabetic eye disease most of the attention has focused on diabetic screening, particularly with regard to uptake, with studies showing the impact of deprivation across the world, including the USA,^14–16^ Canada,^17^ UK,^18–21^ Korea,^22^ India^23^ and Tanzania.^24^ In addition to a lower uptake of screening in the more deprived groups, a number of studies have shown that there are higher rates of sight-threatening diabetic retinopathy (DR) in the most deprived groups.^18 25^ It should be noted that most published data are from screening programmes^18^ or population-based cohort studies,^25^ and there are little data on whether the adverse impact of social deprivation is primarily around screening and access to secondary care, or whether it continues to have an impact once the patient is referred to the hospital eye service.^26 27^ In a small retrospective study from the UK, Lane *et al* 26 found that social deprivation (as measured by the IMD score) was a risk factor for the late presentation of patients with proliferative diabetic retinopathy (PDR) requiring urgent laser therapy, but that care (as measured by time to laser) was not significantly different after entry into the hospital eye service. In a prospective study from the USA, Roy *et al* did not find deprivation to be associated with progression to PDR, although it was associated with incidence of maculopathy.^26 27^ Both studies were relatively small in scale (508 patients or less) and provide little information on progression within secondary care.

The use of electronic medical record (EMR) systems, which routinely gather clinically relevant data, provides the opportunity to analyse larger study cohorts than would be practical within a clinical trial. In the UK, the most widely used ophthalmic EMR has implemented a nationally defined minimum data set for DR that mandates recording of visual acuity status, and the minimum clinical signs necessary to allow automated grading of retinopathy and maculopathy grade after each consultation (as described in the UK DR EMR Reports 1–3).^28–30^ These data therefore enable a large-scale view of the severity of diabetic eye disease at presentation and its rate of progression over time. All interventions including laser procedures, intravitreal injections and ophthalmic operations are recorded, providing the opportunity to analyse access and delivery of treatment. The inclusion of IMD score as part of the core data set now enables us to test in a large ‘real-world’ cohort whether patients with higher levels of deprivation may present later to the hospital eye service in terms of their visual status and their development of sight-threatening complications, and to assess variations in the treatment they receive.

## Methods

### Data collection

Anonymised data were remotely extracted from 22 centres using the same EMR system (Medisoft Ophthalmology, Medisoft, Leeds, UK). Each site is the only National Health Service (NHS) provider of DR care to their local population, and very few patients switch between providers or access care privately. Data were extracted through the EMR compulsory DR structured assessment module. The minimum data set included age, visual acuity at baseline and at all subsequent visits, episodes of intervention (laser, intravitreal therapy, surgical procedure and proxy-ETDRS for retinopathy/International Clinical Grading system grading of maculopathy at baseline and all subsequent visits), and IMD score at baseline.

### Index of Multiple Deprivation

The EMR system includes data on residential address as standard. The LSOA for each postcode was identified and allocated to the IMD score for that area based on the English Indices of Deprivation 2015 (https://www.gov.uk/government/statistics/english-indices-of-deprivation-2015). The LSOA conversion was undertaken at source to avoid transfer of patient-identifiable data. The English Indices of Deprivation 2015 uses the LSOAs defined in the 2011 census, with evaluation of deprivation being primarily based on data taken from 2012 to 2013.

### Inclusion criteria

The study includes eyes from patients with diabetes in whom a structured DR grade(s) and accompanying minimum data set had been recorded in the EMR.

### Analysis

Three cohorts were considered. The ‘all diabetic cohort’ includes all patients who were recorded as diabetic and who had been given a DR grade. It was acknowledged, however, that this would include many ‘low risk’ patients who were referred for reasons other than their diabetic eye disease, for example, cataracts. Since the focus of this study was patients requiring secondary-level care for their diabetic eye disease, we identified two ‘high risk’ cohorts of interest: (1) the ‘early findings cohort’ and (2) the ‘early treatment cohort’. The ‘early findings cohort’ was defined as those patients who were noted to have sight-threatening complications within 2 months of their first visit (comprising treatment-requiring maculopathy, vitreous haemorrhage or tractional retinal detachment). The ‘early treatment cohort’ was defined as those patients who required treatment related to their diabetic eye disease within 2 months of entry into the hospital eye service.

Cohorts were stratified into their IMD deciles (1–10) according to their LSOA as described on https://www.gov.uk/government/statistics/english-indices-of-deprivation-2015. Decile 1 corresponds to the most deprived group. Unless otherwise specified, the eye with worse presenting retinopathy level was chosen for analyses. If both eyes had the same retinopathy level, then one eye was chosen randomly. To provide an internal control and to deal with potential under-reporting from centres which might not use the EMR system beyond basic grading, analysis of sight-threatening complications of diabetic eye disease (diabetic macular oedema, vitreous haemorrhage and tractional retinal detachment) and of treatments was based on the subsets of patients who attended at centres where all relevant conditions and treatments had been recorded.

The primary outcome measure was best corrected visual status (by patient) at entry into the hospital eye service. Visual status was considered in three real-world categories: (1) retained ‘good vision’ in both eyes (better than 6/12 in the worse seeing eye); (2) sight-impaired (visual acuity=6/18 to better than 3/60 in the better seeing eye); and (3) severe sight impairment (3/60 or worse in the better seeing eye). It should be noted that these are not directly equivalent to ‘registration’ grades of sight impairment (previously described as ‘partially sighted’ and ‘blind’) for which qualification may be based on visual acuity alone or a combination of visual acuity and visual field impairment, and which therefore have a degree of subjectivity. Analysis was performed in ETDRS letter notation, using conversions as described for the 1 m LogMAR (logarithm of the minimum angle of resolution) chart as per our previous studies.^28 31 32^

Statistical analysis was done using Ruby (www.ruby-lang.org) and R (www.r-project.org). For each outcome of interest, a logistic regression model was created with the fifth IMD decile set as the reference group.

### Patient and public involvement

This multicentre study evaluating the interaction of deprivation with DR across a broad section of England was directly informed by priorities identified during a James Lind Alliance priority setting partnership.^33^ Priorities identified by this multistakeholder process (including patients and carers) included the need to identify barriers to access in diabetic eye disease and to explore the non-medical factors that lead to differences in outcome. No patients or public were involved in the design of the study or in the numerical analysis. Two patient partners (who wish to remain anonymous) have provided patient commentary of the study, and will be involved in the dissemination of this work through providing patient commentaries to patient societies in both the eyes and vision sector and the diabetes sector for dissemination through written and electronic means.

## Results

### Baseline characteristics

Data were extracted on 79 775 patients (one eye chosen per patient) with DR grades (including a grade of no DR) in the EMR who met the inclusion criteria (patients with diabetes in whom a structured DR grade and minimum data set were recorded). There were 44 646 male patients, 35 127 female patients and 2 cases of unrecorded gender (table 1). At baseline, the study group comprised 8513 eyes with no apparent DR, 43 016 with mild non-proliferativediabetic retinopathy (NPDR), 12 757 with moderate NPDR, 3221 with severe NPDR and 12 268 with PDR. At baseline, 12 581 eyes had maculopathy (any severity).

**Table 1:** Cohort characteristics

	Early findings cohort	Early treatment cohort	All patients with diabetes
n	15 169	6581	79 775
Mean age (SD) (years)	60.60 (13.80)	61.58 (16.64)	63.79 (15.18)
Gender			
Male	9044	3705	44 646
Female	6125	2876	35 127
Unspecified	0	0	2
Mean visual acuity (SD) in the worse seeing eye (number of letters)	67.24 (23.63)	60.47 (28.66)	71.24 (23.04)
Diabetic retinopathy (% eyes)			
No/mild diabetic retinopathy	38.8	36.1	64.6
Moderate NPDR	22.0	5.9	16.0
Severe NPDR	7.8	3.4	4.0
PDR	31.1	54.6	15.4
IMD decile (%)			
1	14.0	18.6	15.9
2	16.3	15.6	15.4
3	13.7	12.9	12.9
4	12.4	10.4	11.2
5	9.1	8.2	8.7
6	8.3	8.4	8.6
7	7.4	7.4	7.7
8	6.8	6.2	6.6
9	7.0	6.8	7.0
10	5.0	5.6	5.8

IMD, Index of Multiple Deprivation; NPDR, non-proliferative diabetic retinopathy; PDR, proliferative diabetic retinopathy.

In the ‘early findings cohort’, there were 15 169 patients who met the inclusion criteria (9044 male patients, 6125 female patients and 0 case of unrecorded gender). In the ‘early treatment cohort, there were 6581 patients who met the inclusion criteria (3705 male patients, 2876 female patients and 0 case of unrecorded gender).

### Visual acuity and visual status at presentation

**Figure 1:**
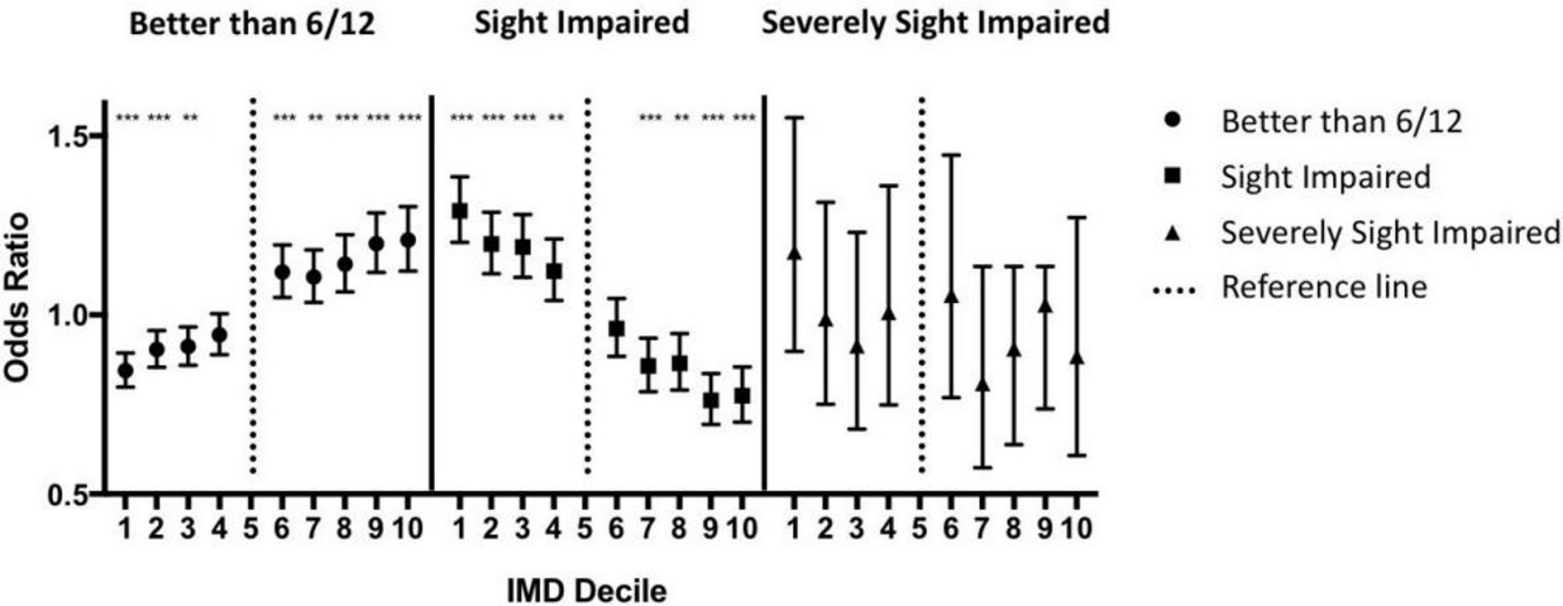
The association of deprivation with worse visual acuity at point of entry into the hospital eye service for patients with diabetic eye disease. Deprivation is plotted per Index of Multiple Deprivation (IMD) decile with the most deprived being decile 1 and the least deprived being decile 10. OR is calculated for each decile in relation to decile 5. The chances of having preserved ‘good vision’ are negatively associated with worse deprivation, whereas the chances of being ‘sight-impaired’ or ‘severely sight-impaired’ are positively associated (see text for definitions). *P<0.05, **p<0.01, ***p<0.001.

Visual acuity at presentation to the hospital eye service was inversely associated with the level of deprivation (figure 1). The mean visual acuity (SD) in the worse seeing eye for the whole diabetic cohort of 79 775 patients was 71.2 (23.0) letters, approximately equivalent to a Snellen score of 6/12-6/11.^31^

Visual status was considered in three real-world categories: (1) retained ‘good vision’ in both eyes (better than 6/12 in the worse seeing eye); (2) sight-impaired (visual acuity=6/18 to better than 3/60 in the better seeing eye); and (3) severe sight impairment (3/60 or worse in the better seeing eye). The OR of having ‘good vision’ was 0.84 (95% CI 0.80 to 0.89) for the most deprived decile vs 1.21 (95% CI 1.12 to 1.30) for the least deprived decile. The OR of being ‘sight impaired’ was 1.29 (95% CI 1.20 to 1.39) for the most deprived decile vs 0.77 (95% CI 0.70 to 0.86) for the least deprived decile; the OR for being ‘severely sight impaired’ (3/60 or worse in the better seeing eye) was 1.17 (95% CI 0.90 to 1.55) for the most deprived decile vs 0.88 (95% CI 0.61 to 1.27) for the least deprived decile (reference=fifth decile in all cases).

### Presence of sight-threatening complications at presentation

**Figure 2:**
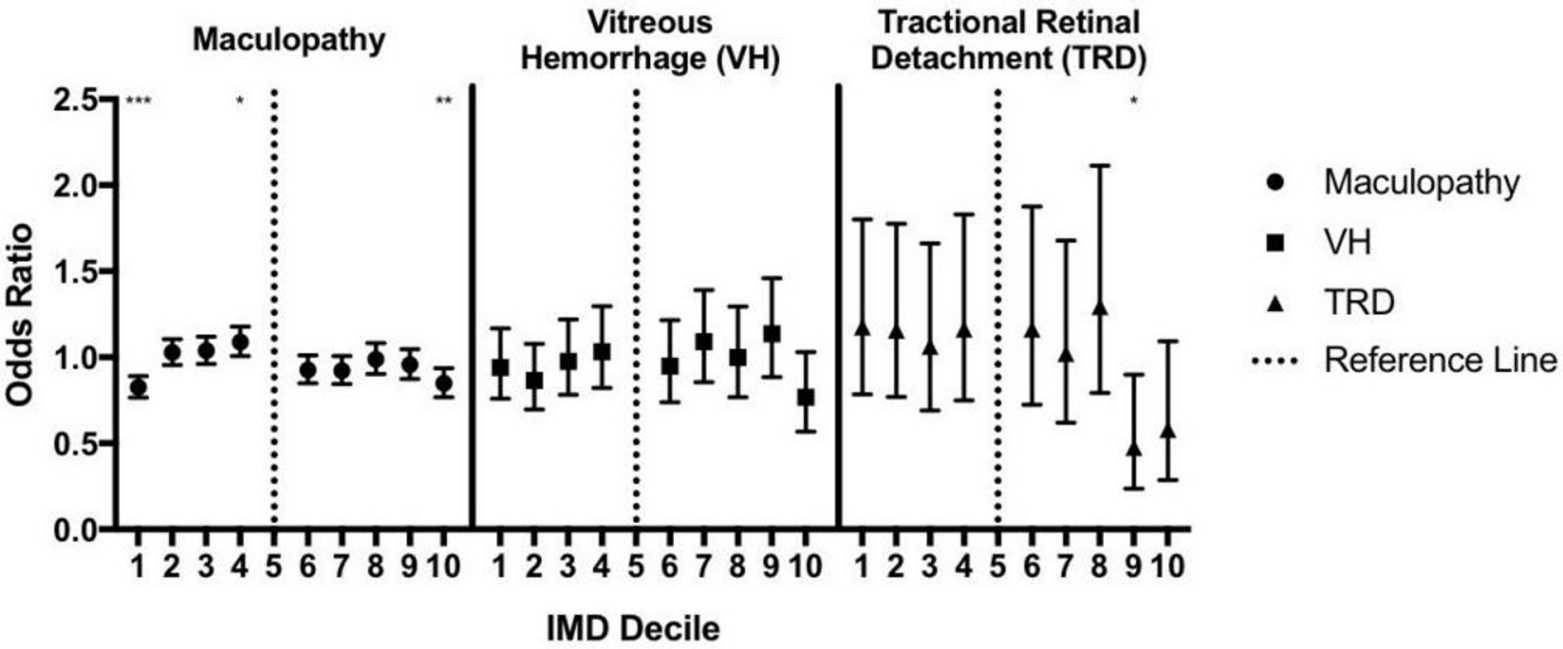
Deprivation and sight-threatening complications of diabetic eye disease. Deprivation is plotted per the Index of Multiple Deprivation (IMD) decile with the most deprived being decile 1 and the least deprived being decile 10. OR is calculated for each decile in relation to decile 5. *P<0.05, **p<0.01.

The ‘early findings cohort’ of 15 169 patients had mean (SD) visual acuities of 67.2 (23.6) ETDRS letters at entry to the hospital service, equivalent to a Snellen score of 6/12-6/15. Nearly 40% of patients in this group had either severe NPDR or PDR. The relationship between deprivation and sight-threatening complications of maculopathy, vitreous haemorrhage and tractional retinal detachment is shown in figure 2.

Maculopathy showed little variation across the IMD deciles, although there was a small but statistically significant reduction in maculopathy in both the most deprived (IMD1) and the least deprived (IMD10) deciles (OR=0.83 (95% CI 0.76 to 0.89 for IMD1); OR=0.85 (95% CI 0.77 to 0.94 for IMD10)). The presence of vitreous haemorrhage was not associated with deprivation level. Tractional retinal detachment was least likely in the deciles with the least deprivation (OR for IMD decile 9, 0.48 (95% CI 0.24 to 0.90); OR for IMD decile 10, 0.58 (95% CI 0.29 to 1.09)).

### Requirement for early treatment for sight-threatening complications

**Figure 3:**
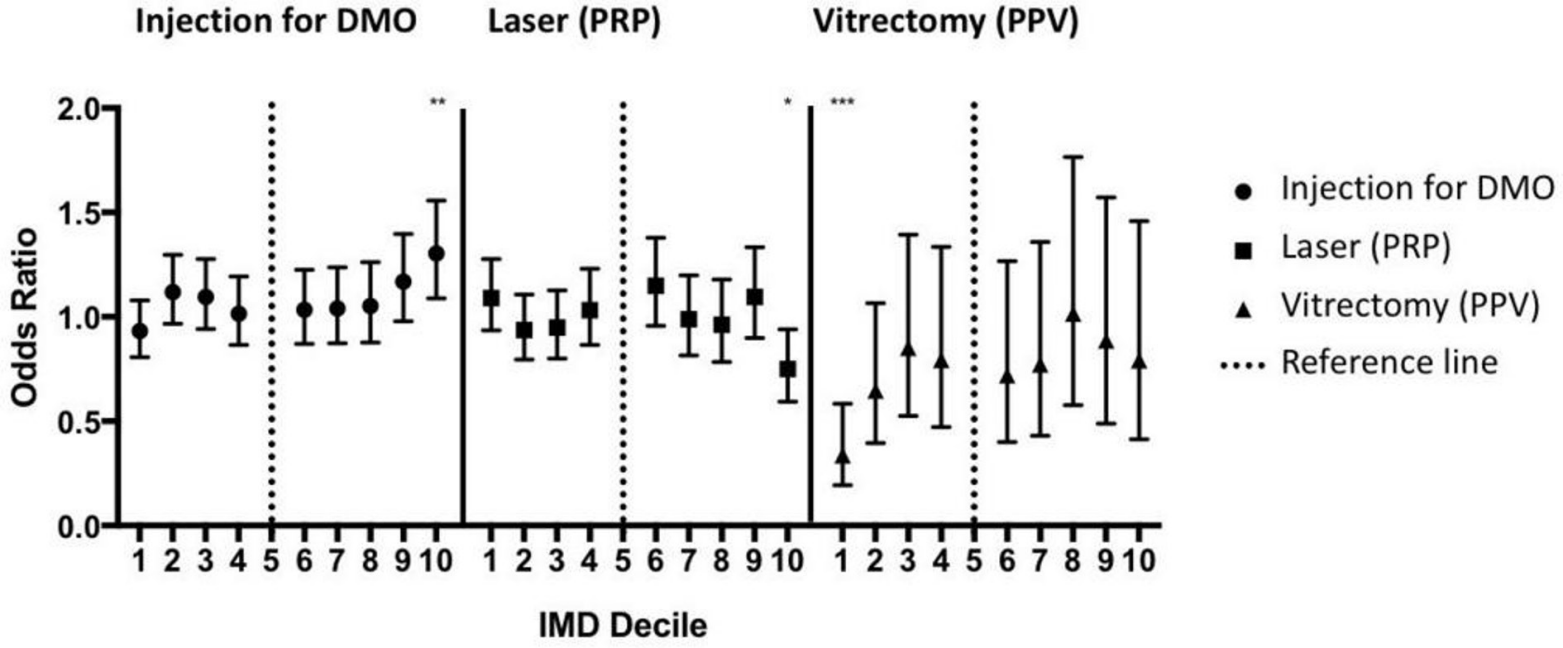
Deprivation and early treatment in diabetic eye disease. Deprivation is plotted per Index of Multiple Deprivation (IMD) decile with the most deprived being decile 1 and the least deprived being decile 10. OR is calculated for each decile in relation to decile 5. Patients in the most deprived decile have a significantly lower chance of having a vitrectomy despite similar rates of vitreous haemorrhage and tractional retinal detachment (compared with the reference). *p<0.05, **p<0.01, ***p<0.001.

The ‘early treatment cohort’ of 6581 patients had a mean (SD) visual acuity of 61.6 (16.6) ETDRS letters at entry to the hospital service, equivalent to a Snellen score of 6/15-6/19. Nearly 60% of patients in this group had either severe NPDR or PDR. The relationship between deprivation and treatment for sight-threatening complications is shown in figure 3.

The strongest association noted was for vitrectomy, which was significantly less likely in the most deprived decile with an OR of 0.34 (95% CI 0.19 to 0.58). Some variation was also noted for both injections for DMO, which was significantly more likely in the least deprived decile (OR=1.3 (95% CI 1.09 to 1.56)), and pan-retinal photocoagulation, which was significantly less likely (OR=0.75 (95% CI 0.59 to 0.94)).

## Discussion

Deprivation is a major determinant of health, being shown to be associated with late disease presentation and worse outcomes across a range of health problems. This analysis of over 75 000 patients with diabetes taken from across 22 centres across England shows that the impact of deprivation extends to late presentation of diabetic eye disease. At the time of presentation to the hospital eye service, the likelihood of a patient with diabetes having lost significant vision (‘sight impaired’) is much higher in the most deprived decile (OR 1.29) than the least deprived decile (OR 0.77; both vs the fifth decile as a reference). There also appears to be an interaction with the patterns of sight-threatening complications seen (such as tractional retinal detachment) and the treatment that these patients receive.

Most larger scale studies of the effect of deprivation on diabetic eye disease do suggest an association, although it should be noted that there are important differences in the design and setting. First most are based on community screening data and not conducted within the hospital eye service. In the UK, Scanlon *et al* 18 used IMD data to analyse the effect of deprivation on over 13 000 patients seen as part of a national diabetes retinopathy screening programme. They did not find higher levels of deprivation to be associated with a higher prevalence of retinopathy per se but found that deprivation was associated with higher levels of sight-threatening retinopathy, which increased from 11.9% in quintile 1 (least deprived) to 14.2% in quintile 5 (most deprived). In the USA, West*et al* 25 reported on 4774 Hispanics aged 40 years and over, compiled as part of the Proyecto VER Study. In this high-risk group the presence of diabetes was associated with deprivation as measured by lower income and lower educational attainment, and the presence of PDR was associated with low income (OR=3.6, for developing PDR if income <$20 000) after controlling for other factors.^25^

A second important difference in our analysis presented here is that our primary outcome is an outcome that is meaningful to patients—visual acuity or binocular visual status—rather than retinopathy grade per se. Visual status at the time of entry into the hospital eye service was considered in three real-world categories: ‘good vision’ in both eyes (sufficient for reading or driving), ‘sight impaired’ and ‘severely sight-impaired’. This is not to underestimate the importance of retinopathy grade, but to acknowledge that the importance of retinopathy is related to its functional impact on the patient (ie, loss of vision). The relationship of retinopathy to functional outcome is complex, but we are continuing to explore this in this cohort, using both the cross-sectional and longitudinal data available.

It is notable that a number of smaller studies have failed to detect an effect between deprivation and diabetic eye disease, but report a non-significant trend. We therefore contend that the failure to detect an effect of deprivation likely arose due to a lack of statistical power. For example, the study by Lim *et al* 34 of 1073 patients from the San Francisco mobile eye service noted that the level of severe retinopathy was more than twofold higher in the lowest income group, but this did not reach statistical significance. Some variation between the findings of studies may also be attributable to how deprivation has been assessed, with a number of studies assessing deprivation on only educational or financial criteria.^35^

The major strengths of our study include its scale, the use of a comprehensive deprivation assessment tool and its longitudinal data. The scale of our study, which includes 79.775 eyes, is made possible by its ‘real world’ nature arising from the use of a standardised assessment framework within the most widely used ophthalmic EMR system in the UK. Our study includes data from 22 different centres from across the UK, which include urban and rural settings, with a wide range of ethnic and demographic profiles, providing a broader analysis of the situation in the UK than was possible in the single-area study by Scanlon *et a* *l*.^18^ One of the great strengths of both the Scanlon study and ours is the use of the IMD tool to measure deprivation. The multiple components of the IMD tool provide a more complete assessment of deprivation than a simple assessment of income or level of educational attainment. The IMD tool includes assessments of income, employment, health and disability, education, crime, barriers to housing, services and living environment.

In contrast to most studies in this area which are cross-sectional in design, our study provides in-hospital data to evaluate treatment decisions. This has highlighted that in those patients who required treatment for sight-threatening complications of diabetes within 2 months of first hospital visit (the ‘early treatment group’), there appears to be some variation in practice at the extreme ends of the deprivation spectrum. It is interesting to note that pars plana vitrectomy is significantly less common in the most deprived decile, despite having similar rates of both vitreous haemorrhage and tractional retinal detachment as most other deciles; indeed it is the least deprived deciles that appear to be relatively protected from presenting with tractional retinal detachment in the electronic record. Injection treatment for diabetic maculopathy was significantly more common in the least deprived decile, even though diabetic maculopathy was recorded significantly less commonly in this decile.

Although these differences in treatment pattern could reflect an underlying degree of inequity between groups of patients, the association is likely to be complex. The low rates of vitrectomy in the most deprived cohorts could represent one or more of the *disease factors*—more advanced disease at presentation which is deemed to be beyond surgical repair; *concomitant medical factors* (eg, diabetes control, renal failure, uncontrolled hypertension) or *social factors* (employment, childcare responsibilities) that may have delayed treatment delivery beyond the 8-week limit used in this analysis; *surgeon choice* including justifiable understanding of risk and unjustified biases; and *patient choice* including variations in understanding risk, medical jargon. It is also possible that there is some effect from the less deprived deciles receiving part of their care in the private sector, although we believe that this is likely to be relatively minor given the distribution of NHS versus private care in the UK.^36^ These are important questions which would require more indepth qualitative work. Our study can however highlight these issues as being worthy of further investigation.

In the future, longitudinal data from our study will also enable us to evaluate whether the effect of deprivation continues after entry into the hospital eye service. We are now exploring the relationship between IMD score and progression of retinopathy to provide an estimate of rates of progression and decline in visual acuity for patients with diabetes within the hospital eye service, which may be directly useful to clinical practice and service provision. Further analysis may enable early identification of high-risk groups, stratification of frequency of follow-up and more targeted intervention to avoid the development of blindness in those at highest risk.

While our use of the IMD tool and the ‘real world’ EMR-based design has considerable advantages, we recognise that they are not without their challenges and limitations. The IMD tool is based on geographical area, which means that deprivation is defined by ‘neighbourhood’ and not by household or by the individual. Under some circumstances individuals may live in a neighbourhood that is somewhat different from their own deprivation status. This risk is however reduced by the small size of the areas used (LSOA). Since 2000 the UK government has found the IMD tool to be a reliable and valuable measure of deprivation within England, with good internal and external validity justifying its ongoing usage.^37^

The main challenge arising from most ‘real world’ studies such as ours is the quality assurance of data. While we recognise that quality assurance may not be of the same level as a randomised controlled trial, there are a number of characteristics of our study that provide reassurance in this regard. First, the EMR design ensures that the data entry is structured, with key fields being compulsory and low rates of missing non-compulsory fields.^32^ Second, data fields have value cut-offs to stop major data entry errors; most minor errors may occur in either direction and any effect is minimised by the large size of the study. Overall we would argue that we achieve a high ‘signal:noise’ ratio in this real-world study through the design of the underlying data capture tool (through the EMR system) and the scale of the study incorporating a large number of patients over many different centres across the UK.^28–30^

In summary, this analysis of a real-world data set from a large number of centres across the UK indicates an association that those people with diabetes and higher levels of deprivation are more likely to present to the hospital eye service with worse visual acuity and binocular visual status. The presence of this ‘deprivation effect’ on the level of vision at the first point of contact with a specialist eye service is a concern. We also identified an apparent interaction between pattern of treatment for sight-threatening complications of diabetes and level of deprivation.

It is important to note that our analysis was conducted within a universal ‘free’ system (the UK NHS) and that this effect may be more pronounced in countries where there is differential access to healthcare based on ability to pay. The recognition of a ‘deprivation effect’ on diabetic eye disease within different societies may help policy makers direct appropriate interventions and resources to the most vulnerable, and help reduce the inequality of outcome.

10.1136/bjophthalmol-2018-312568.supp1 Supplementary data
