# Supplementary material for: United Kingdom Diabetic Retinopathy Electronic Medical Record (UK DR EMR) Users Group: report 4, real-world data on the impact of deprivation on the presentation of diabetic eye disease at hospital services
Source: Br J Ophthalmol. 2018 Sep 29;103(6):837–43. doi: 10.1136/bjophthalmol-2018-312568 (PMC6582816; doi:10.1136/bjophthalmol-2018-312568)
Supplement: Supplementary data [file bjophthalmol-2018-312568supp001.pdf]

Appendix 1:

Sites from the UK DR EMR Users Group contributing to Report 4

NHS Trust Site	Investigator
Barking, Havering and Redbridge University Hospitals NHS Trust	Bobby Paul
Bart's Health NHS Trust	Sudeshna Patra
Belfast Health and Social Care Trust	Usha Chakravarthy
Bradford Teaching Hospitals NHS Foundation Trust	Faruque Ghanchi
Calderdale and Huddersfield NHS Foundation Trust	Rehna Khan
Frimley Park Hospital NHS Foundation Trust	Geeta Menon
Hinchingbrooke Health Care NHS Trust	Toks Akerele
Hull and East Yorkshire Hospitals NHS Foundation Trust	Louise Downey
King's College Hospital NHS Foundation Trust	Haralabos Eleftheriadis
Leeds Teaching Hospitals NHS Trust	Raj Mukherjee
Mid Yorkshire Hospitals NHS Trust	Narendra Dhingra
Moorfields Eye Centre at Bedford Hospital	Aires Lobo
Moorfields Eye Centre at Croydon University Hospital	Dawn Sim
Northern Devon Healthcare NHS Trust	Elizabeth Wilkinson
Royal United Hospital Bath NHS Trust	Richard Antcliff
Sandwell and West Birmingham Hospitals NHS Trust	Randhir Chavan
Sheffield Teaching Hospitals NHS Foundation Trust	Chris Brand
The Newcastle Upon Tyne Hospitals NHS Foundation Trust	James Talks
University Hospital Southampton NHS Foundation Trust	Andrew Lotery
University Hospitals Birmingham NHS Foundation Trust	Helen Palmer
University Hospitals Bristol NHS Foundation Trust	Clare Bailey
Wirral University Teaching Hospital NHS Foundation Trust	Vineeth Kumar
